# Molecular diversity of *Mycobacterium tuberculosis* complex in Sikkim, India and prediction of dominant spoligotypes using artificial intelligence

**DOI:** 10.1038/s41598-021-86626-z

**Published:** 2021-04-01

**Authors:** Kangjam Rekha Devi, Jagat Pradhan, Rinchenla Bhutia, Peggy Dadul, Atanu Sarkar, Nitumoni Gohain, Kanwar Narain

**Affiliations:** 1 N.E. Region, Indian Council of Medical Research (ICMR)-Regional Medical Research Centre, Post Box #105, Dibrugarh, Assam 786 001 India; 2 National Tuberculosis Elimination Programme (NTEP), Gangtok, Sikkim India; 3 Department of Health Care, Human Services and Family Welfare, State Tuberculosis Control Society, Gangtok, Sikkim India

**Keywords:** Bacteriology, Microbial communities

## Abstract

In India, tuberculosis is an enormous public health problem. This study provides the first description of molecular diversity of the *Mycobacterium tuberculosis* complex (MTBC) from Sikkim, India. A total of 399 Acid Fast Bacilli sputum positive samples were cultured on Lőwenstein–Jensen media and genetic characterisation was done by spoligotyping and 24-loci MIRU-VNTR typing. Spoligotyping revealed the occurrence of 58 different spoligotypes. *Beijing* spoligotype was the most dominant type constituting 62.41% of the total isolates and was associated with Multiple Drug Resistance. Minimum Spanning tree analysis of 249 *Beijing* strains based on 24-loci MIRU-VNTR analysis identified 12 clonal complexes (Single Locus Variants). The principal component analysis was used to visualise possible grouping of MTBC isolates from Sikkim belonging to major spoligotypes using 24-MIRU VNTR profiles. Artificial intelligence-based machine learning (ML) methods such as Random Forests (RF), Support Vector Machines (SVM) and Artificial Neural Networks (ANN) were used to predict dominant spoligotypes of MTBC using MIRU-VNTR data. K-fold cross-validation and validation using unseen testing data set revealed high accuracy of ANN, RF, and SVM for predicting Beijing, CAS1_Delhi, and T1 Spoligotypes (93–99%). However, prediction using the external new validation data set revealed that the RF model was more accurate than SVM and ANN.

## Introduction

In India, the burden of tuberculosis (TB) is enormous. According to the latest estimate of the World Health Organisation (WHO), the largest number of incident cases in 2018 were from India (2.69 million, 95% CI 1.84 to 3.70 million) accounting for 27% of global cases^1^. Advances in molecular technology have helped us understand the genetic structure of *Mycobacterium tuberculosis* complex (MTBC) providing insights regarding the population dynamics and spread of MTBC locally and globally. The information obtained by molecular typing of MTBC isolates is essential for understanding TB epidemics and preventing TB^2–4^. Current studies also indicate that the outcome of TB infection may be related to strain diversity of MTBC^5,6^. For example, "*Beijing* strain" of MTBC has been reported to be more virulent in animal models and is often reported to be responsible for causing outbreaks^7,8^. Moreover, knowledge of the genetic diversity of MTBC is very useful for assessing the impact of the TB control program^9^. Although numerous studies on the genetic diversity of MTBC have been conducted in India^9–41^ yet no such types of studies are available from the hill state of Sikkim where the prevalence of MDR strains of MTBC is high^42^.

Sikkim is a small hilly state in the North East region of India, adjacent to three neighbouring countries like China, Nepal, and Bhutan. India TB report showed a high incidence of TB cases^43^. To better understand the genetic diversity of multidrug-resistant (MDR) and Non-MDR MTBC circulating in Sikkim, we characterized 399 MTBC isolates from Sikkim using spoligotyping and 24-loci Mycobacterial Interspersed Repetitive Unit-Variable Number of Tandem Repeats (MIRU-VNTR) typing. Spoligotyping is a PCR-based reverse-hybridization blotting technique based on polymorphisms in the presence or absence of "spacers" in the Direct Repeat (DR) locus of MTBC^44^. Typing of MTBC using spoligotyping led to the creation of the database "SpolDB4" in 2006 which gave the first overview into the global diversity and phylogeography of MTBC spoligotypes^44^. Subsequently, SITVIT web & SITVIT2 databases were created.

A more robust genotyping method, namely 24-loci MIRU-VNTR typing is being used for the genotypic characterization of MTBC isolates. Numerous studies have shown that 24-loci MIRU-VNTR genetic markers have high discriminatory power, provide deep insight into MTBC Spoligotypes and sub-Spoligotypes and thus can be used as a very good alternative method for IS6110 restriction fragment length polymorphism (RFLP) which has numerous limitations^45–48^. Our study aimed to understand the genetic diversity of clinical isolates of MTBC from pulmonary tuberculosis cases from Sikkim a remote state in North-eastern India where the burden of tuberculosis is an emerging public health concern.

Spoligotyping of MTBC strains is widely used in the epidemiological studies on tuberculosis^49^. However, due to technical difficulties and possibility of carryover contamination of the hybridization membrane, numerous researchers have developed alternative methods like mass spectrometry^50^, Luminex MagPlex magnetic microspheres^51^, multicolour melting curve analysis^52^ for carrying out spoligotyping. In this study we tried to predict dominant spoligotypes prevalent in Sikkim using 24-loci MIRU-VNTR profile using Artificial Intelligence.

## Results

### Spoligotyping

**Figure 1:**
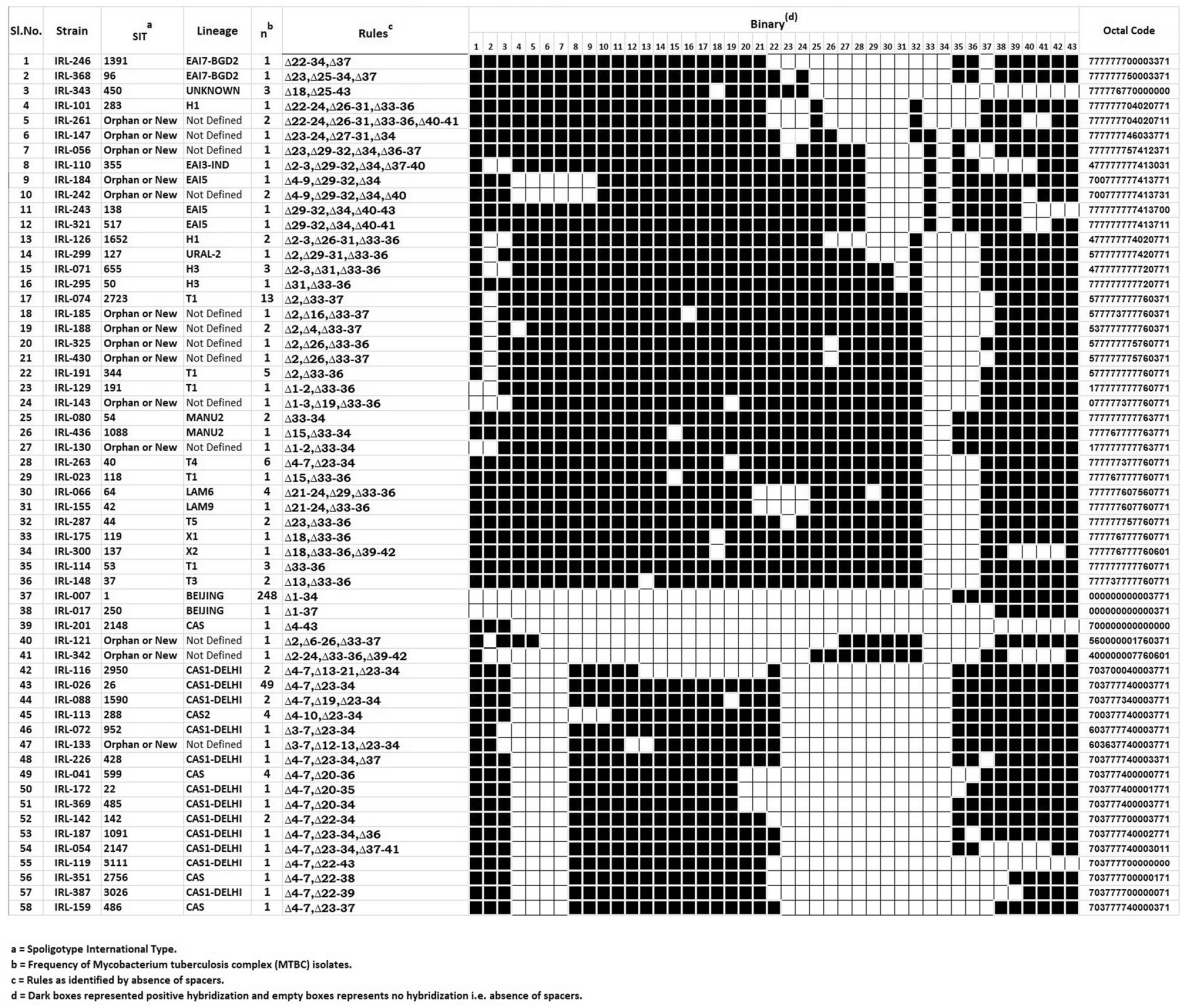
58 patterns of spoligotypes of MTBC present in Sikkim (2016–2018). The spoligotype patterns are made in Microsoft Excel2019 (https://www.microsoft.com/en-in). We used Microsoft Windows Screenshot Snipping Tool to save as image (https://support.microsoft.com/en-in/help/13776/windows-10-use-snipping-tool-to-capture-screenshots).

**Figure 2:**
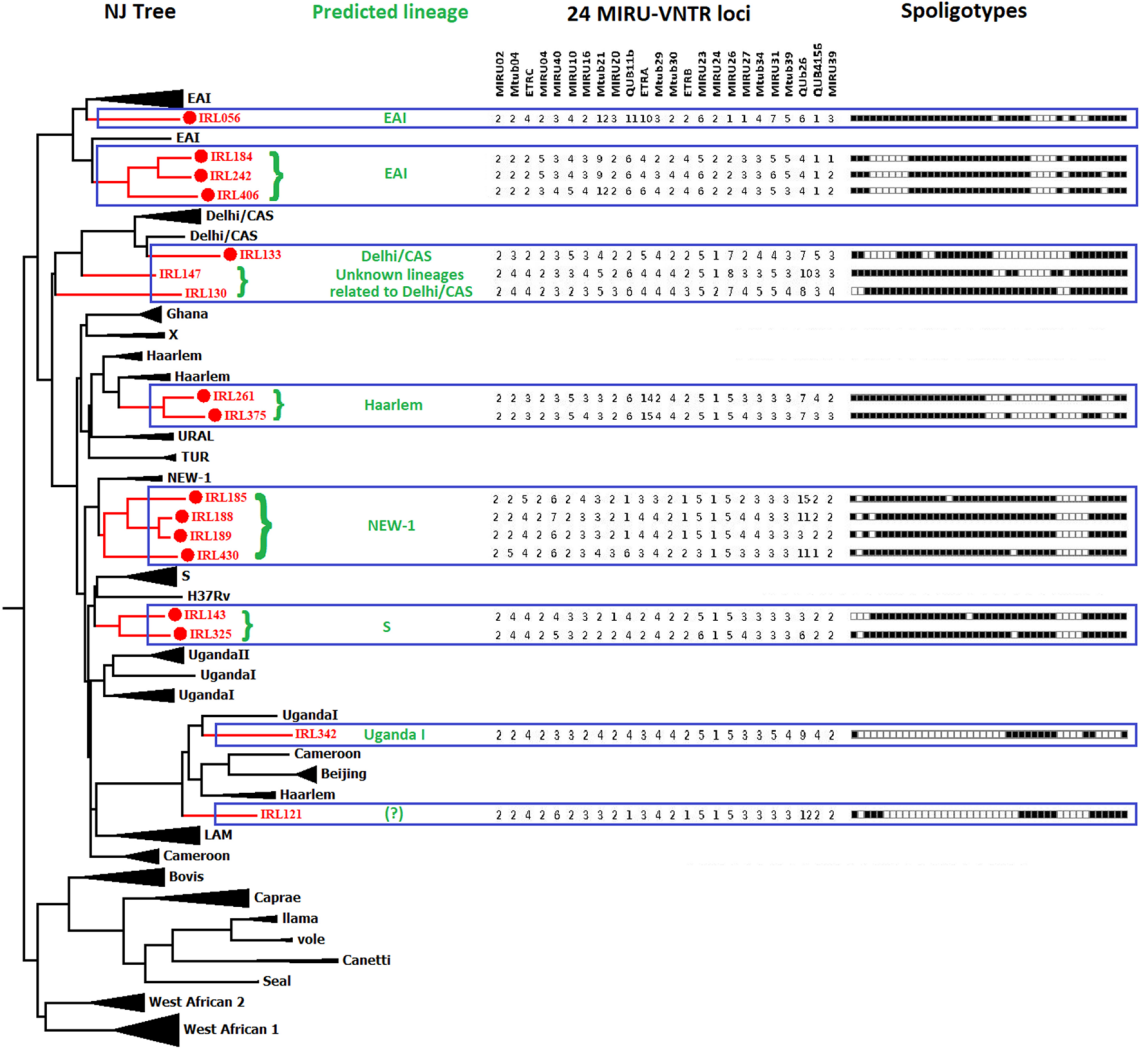
Neighbor-Joining (NJ) tree showing the phylogenetic relationship of orphan strains of MTBC from Sikkim with reference MTBC isolates available at the MIRU-VNTR*plus* database. The NJ tree was constructed using spoligotyping and 24-loci MIRU-VNTR data. MIRU-VNTR alleles and spoligo-patterns from 17 isolates are also represented along with the NJ tree. This phylogenetic tree was used to predict lineage of the orphan/new MTBC isolates from Sikkim.Web tools MIRU-VNTR*plus* (https://www.miru-vntrplus.org) and MEGA v7.0 were used to make the phylogenetic trees (https://www.megasoftware.net).

The 399 MTBC isolates from Sikkim were found to be representing 58 different spoligotypes shown in Fig. 1. Distribution of different Spoligotypes of MTBC spoligotypes isolated from Sikkim in the study based on the classification by SITVIT2 Web is given in Table 1. Spoligotype International Types (SIT) Beijing/SIT1/SIT250 with 249 isolates was the most dominant type (62.41%, n = 399) followed byCAS1_Delhi/SIT2950/SIT26/SIT1590/SIT952/SIT428/SIT22/SIT485/SIT142/SIT1901/SIT2147/SIT3111/SIT3026 with 63 isolates (15.79%, n = 399), T1/SIT2723/SIT334/SIT191/SIT118/SIT53 with 23 isolates (5.76%, n = 399), CAS/SIT2148/SIT599/SIT2756/SIT486 with 7 isolates (1.75%, n = 399), T4/SIT40 with 6 isolates (1.50%, n = 399), CAS2/SIT288, H3/SIT665/SIT50, LAM6/SIT64 with 4 isolates (1.00%, n = 399), EAI5/SIT138/SIT517, H1/SIT283, MANU2/SIT54/SIT1088, UNKNOWN/SIT450 with 3 isolates (0.75%, n = 399), EAI7-BGD2/SIT1391/SIT96, T3/SIT37, T5/SIT44 with 2 isolates (0.50%, n = 399). The other known spoligotypes were unique and represented by 1 isolate of EAI3-IND/SIT355, LAM9/SIT42, URAL-2/SIT127, X1/SIT119 and X2/SIT137, (0.25%, n = 399). In our study, 14 spoligotypes were found to be new (not found in SITVIT2 database), out of which 11 patterns were orphans (from single patients), and the remaining 3 spoligopatterns were new SITs (present in 6 patients). Out of 14 new spoligotypes 13 were genetically close to Delhi/CAS, NEW1, EAI, S, Haarlem, and Uganda l spoligotypes based on Neighbor-Joining phylogenetic tree analysis including reference spoligotype database (Table 2, Fig. 2 and Supplementary Fig. 1). However, one new spoligotype could not be predicted.

**Table 1:** Distribution of different lineages of 399 MTBC isolates collected from 2016 to 2018 based on the spoligotype classification by SITVIT2.

Lineage	No. of isolates	Prevalence*
BEIJING	249	62.41
CAS1-DELHI	63	15.79
T1	23	5.76
Orphan	17	4.26
CAS	7	1.75
T4	6	1.50
CAS2	4	1.00
LAM6	4	1.00
H3	4	1.00
MANU2	3	0.75
EAI5	2	0.50
H1	3	0.75
UNKNOWN	3	0.75
T3	2	0.50
T5	2	0.50
EAI7-BGD2	2	0.50
X2	1	0.25
X1	1	0.25
LAM9	1	0.25
URAL-2	1	0.25
EAI3-IND	1	0.25

*Values are in %.

**Table 2:** Predicted lineages of 17 orphan MTBC* isolates using phylogenetic tree-based identification as implemented by MIRU-VNTRplus.

Sample ID of New spoligotypes discovered**	n	Predicted lineage
IRL-056	1	EAI
IRL-121	1	?
IRL-130	1	Delhi CAS
IRL-133	1	Delhi CAS
IRL-143	1	S
IRL-147	1	Delhi CAS
IRL-185	1	NEW1
IRL-188 & IRL-189	2	NEW1
IRL-242 & IRL-406	2	EAI
IRL-261 & IRL-375	2	Haarlem
IRL-325	1	S
IRL-342	1	Uganda I
IRL-430	1	NEW1
IRL-184	1	EAI

*Mycobacterium tuberculosis complex.

**Total number of patterns: 14.

In the present study, 362 (90.7%, n = 399) isolates occurred in clusters. The Hunter Gaston Discriminatory Index of spoligotyping was low (HGDI = 0.5977) in MTBC of Sikkim (Table 3).

**Table 3:** Hunter Gaston Discriminatory Index (HGDI) and cluster results based on MIRU-VNTR loci analysis of 399 Mycobacterium tuberculosis complexes (MTBC) isolate from Sikkim.

Typing method	Total no. of patterns	No. of unique types	Total no. of clusters	Total no. of isolates in clusters*	Maximum no. of isolates in a cluster	HGDI
Spoligotyping	58	37	21	362 (90.7)	248	0.5977
24 loci MIRU-VNTR	394	389	5	10 (2.51)	2	0.999937029

*Values are in %.

### MIRU-VNTR typing

Table 4 summarizes the diversity of the 24-loci MIRU-VNTR in MTBC isolates from Sikkim. Analysis of allelic diversity of 24-loci MIRU-VNTR revealed that out of 24-loci, 9 loci (Mtub04, MIRU10, MIRU16 Mtub21, QUB11b, MIRU26, MIRU31, QUb26, QUB4156, MIRU39) showed high discriminatory power (above or equal to 0.6). Allelic diversity of 24-loci MIRU-VNTR in Sikkim based on stratified analysis of *Beijing* and Non-*Beijing* strains revealed that discriminatory power of various MIRU-VNTR alleles was lower in *Beijing* isolates as compared to Non-*Beijing* isolates. Alleles such as (MIRU02, Mtub04, ETRC, MIRU04, MIRU40, MIRU10, MIRU16, MIRU20, QUB11b, ETRA, Mtub29, Mtub30, ETRB, MIRU23, MIRU26, Mtub34, Mtub39, QUb26, QUB4156, MIRU39) showed lower discriminatory power in *Beijing* MTBC isolates from Sikkim as compared to Non-*Beijing* isolates from Sikkim.

**Table 4:** The diversity of each of the 24 MIRU-VNTR loci in Beijing (n = 249) and Non-Beijing (n = 150) Mycobacterium tuberculosis isolates from Sikkim.

Alias	Locus	HGDI		
		Beijing	Non-Beijing	Total
MIRU02	154	0.1713	0.9497	0.1896
Mtub04	424	0.6071	0.7264	0.6930
ETRC	577	0.2190	0.6375	0.4740
MIRU04	580	0.0080	0.1769	0.0736
MIRU40	802	0.3653	0.6145	0.4702
MIRU10	960	0.3157	0.8438	0.6228
MIRU16	1644	0.6242	0.6486	0.6444
Mtub21	1955	0.5326	0.7116	0.6540
MIRU20	2059	0.2908	0.3238	0.3033
QUB11b	2163b	0.4502	0.7221	0.6830
ETRA	2165	0.1083	0.6801	0.3975
Mtub29	2347	0.3326	0.3480	0.3383
Mtub30	2401	0.2171	0.5000	0.5330
ETRB	2461	0.2092	0.5155	0.3407
MIRU23	2531	0.1137	0.3537	0.2099
MIRU24	2687	0.3691	0.3661	0.3672
MIRU26	2996	0.6901	0.7941	0.7539
MIRU27	3007	0.4289	0.4756	0.4464
Mtub34	3171	0.2476	0.4045	0.3087
MIRU31	3192	0.6969	0.7536	0.7597
Mtub39	3690	0.3967	0.5486	0.4566
QUb26	4052	0.7078	0.8774	0.7914
QUB4156	4156	0.6917	0.7652	0.7210
MIRU39	4348	0.5346	0.6795	0.6170

The 399 MTBC isolates from Sikkim were found to represent 394 24-loci MIRU-VNTR profiles out of which 389 profiles were unique, i.e. each type is represented by only one MTBC isolate and 5 MIRU-VNTR types formed clusters and the clustering rate was 2.51% (Table 3). The maximum number of isolates in a cluster was 10. The Hunter-Gaston Discriminatory Index (HGDI) of combined 24-loci MIRU-VNTR typing analysis was 0.9999.

To capture population snapshot of genetic diversity of Beijing and Non-Beijing MTBC isolates from Sikkim we used Minimum Spanning Tree analysis using 24-loci MIRU-VNTR data. We also determined the presence of Clonal Complexes based on Single Locus Variants (SLVs) i.e. MTBC isolates having similar MIRU-VNTR profiles but differ only at a single locus. Neighbor-Joining phylogenetic tree of 249 Beijing and 150 Non-Beijing MTBC isolates from Sikkim based on spoligotyping data and 24-MIRU-VNTR profile is also given (Supplementary Fig. 2 and 3).

**Figure 3:**
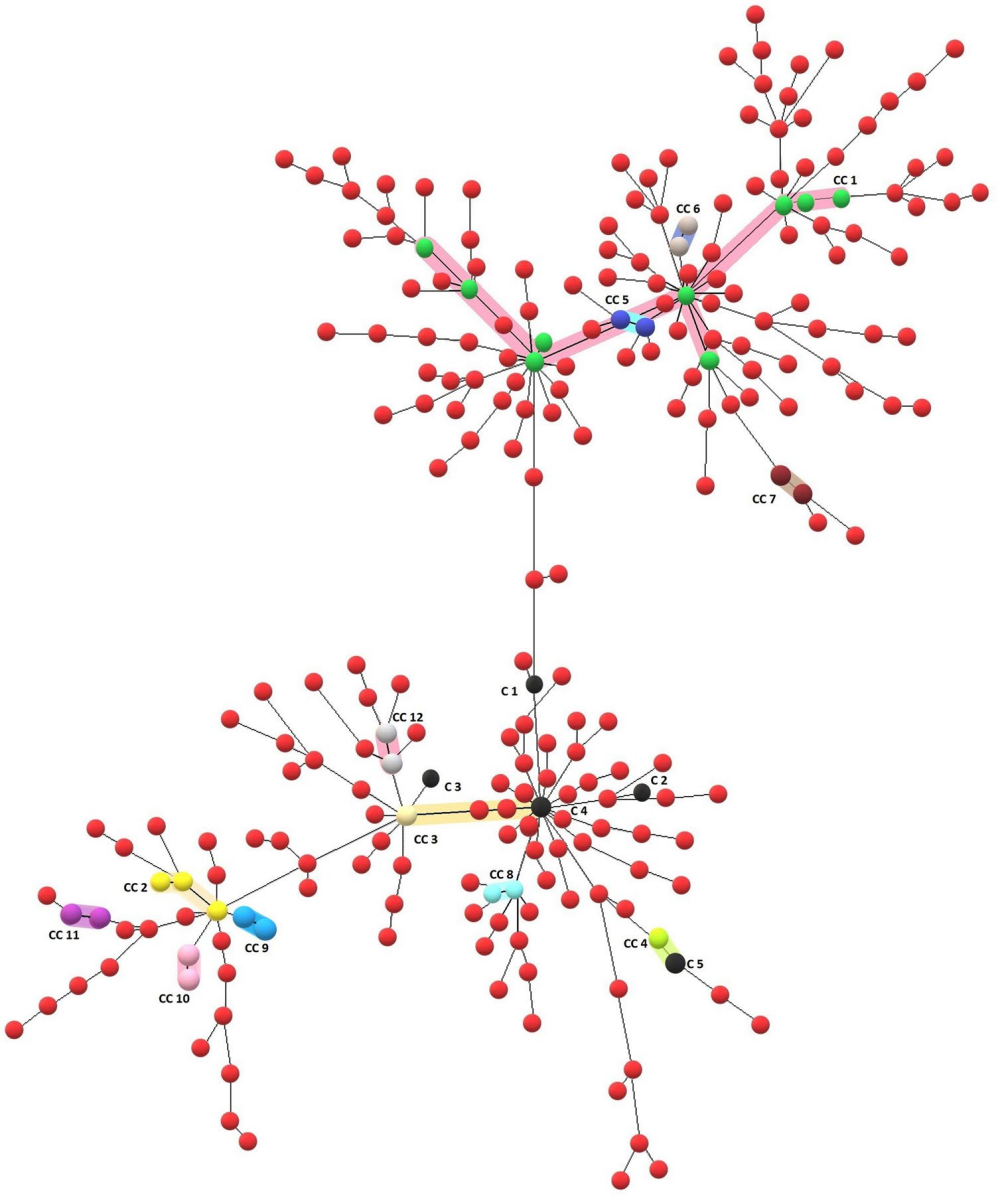
A Minimum Spanning Tree (MST) depicting relationships among 249 Beijing isolates from Sikkim, India based on 24-loci MIRU-VNTR data. This tree shows the clustering of MTBC isolates based on a final match of 24-loci MIRU-VNTR profiles (10 isolates represented by 5 black dots) and clusters based on Single Locus Variant (SLV-1) (i.e. these clusters of isolates which differ from each other by the single difference in 24-loci MIRU-VNTR profile). The largest Clonal Complex (CC1) comprisesnine isolates shown by green dots and highlighted in pink colour. This MST was developed using the MIRU-VNTRplus web tool (https://www.miru-vntrplus.org) and the figure was enhanced using Microsoft Paint (https://ms-paint.en.softonic.com).

**Figure 4:**
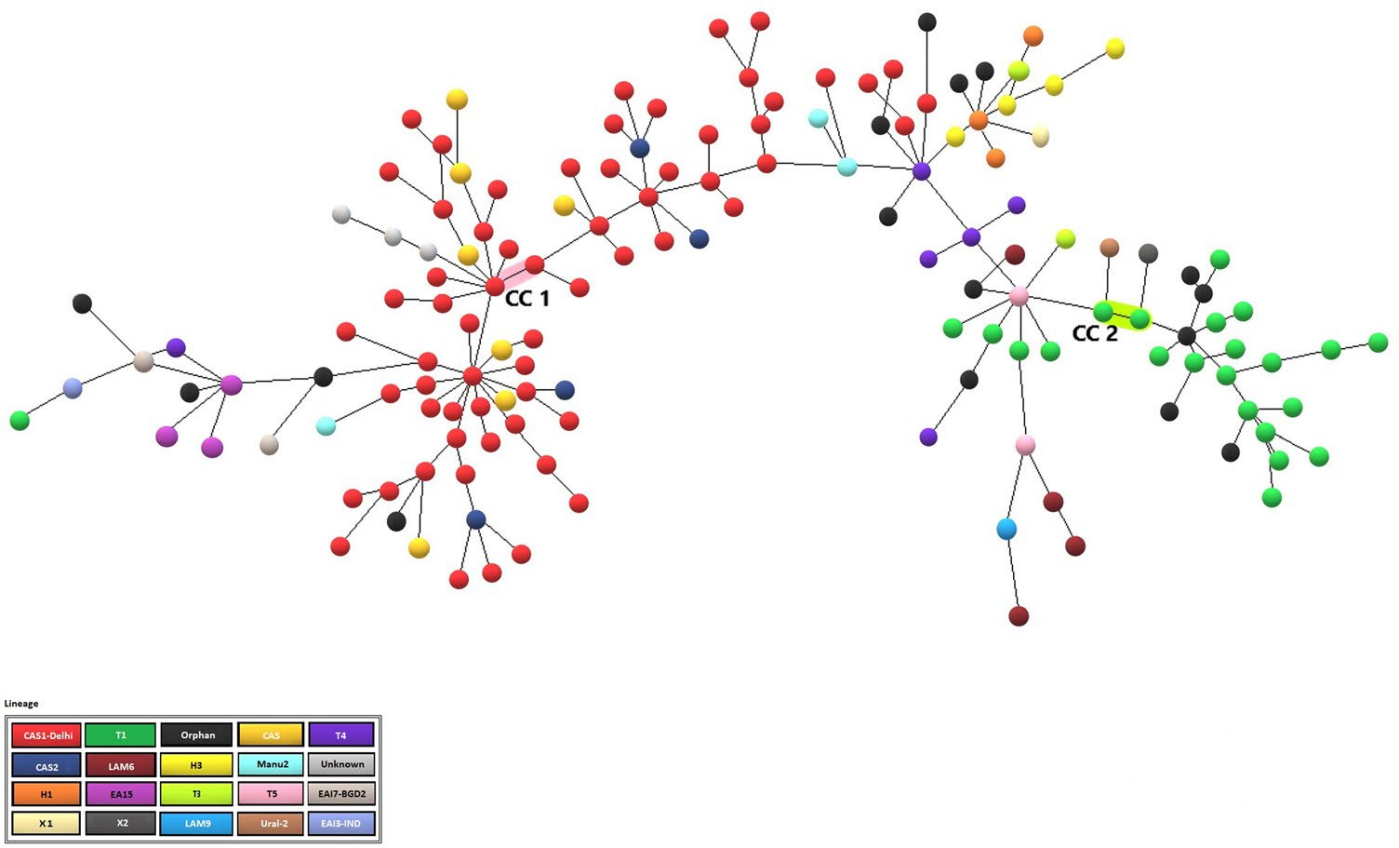
The Minimum Spanning Tree (MST) of 150 Non-Beijing isolates from Sikkim, India. Only two Clonal Complexes (CC1 & CC2) are present. The most dominant lineage among non-Beijing isolates was CAS1-Delhi represented by red dots in the MST. 17 orphan/new MTBC isolates discovered in the study are represented by black dots. This MST was developed using the MIRU-VNTRplus web tool (https://www.miru-vntrplus.org), and the figure was enhanced using Microsoft Paint (https://ms-paint.en.softonic.com/).

Out of 249 Beijing isolates, 34 (13.6%) isolates were distributed in 12 Clonal Complexes (CCs), 10 isolates formed 5 identical clusters that are having identical MIRU-VNTR profiles, and the remaining 205 (82.3%) isolates were unique. The largest clonal complexes (CC1) and (CC2) include 9 and 3 isolates, respectively (Fig. 3). On the other hand, a 24-locus MIRU-VNTR based MST for Non-Beijing isolates could identify only two clonal complexes and these CCs (CC1 & CC2) included 2 isolates each (Fig. 4).

### Prevalence of Multiple Drug Resistance according to spoligotypes

Out of 249, *Beijing* isolates 74 (29.7%) were Multiple Drug Resistant (MDR) in contrast to 7 (4.7%) out of 150 Non-*Beijing* isolates. *Beijing* isolates had more than 8.6 times higher risk of being MDR (Odds ratio 8.64; 95%. CI 3.86–19.34; *p* ≤ 0.01) than Non-*Beijing* strains and this difference was statistically significant (Table 5).

**Table 5:** Results of multiple logistic regression analysis showing the association of Beijing isolates of MTBC with multiple drug resistance (MDR). The dependent variable was MDR, and the independent variable was Beijing and non-Beijing strains of MTBC.

	Coefficient	Standard error	chi-square	df*	Significance	Odds ratio	C.I**. for odds ratio	
							Lower	Upper
Beijing(1)	2.156	0.411	27.499	1	0	8.638	3.859	19.339
Constant	− 3.017	0.387	60.74	1	0	0.049		

*Degree of freedom; **95% Confidence Interval.

### Principal component analysis

**Figure 5:**
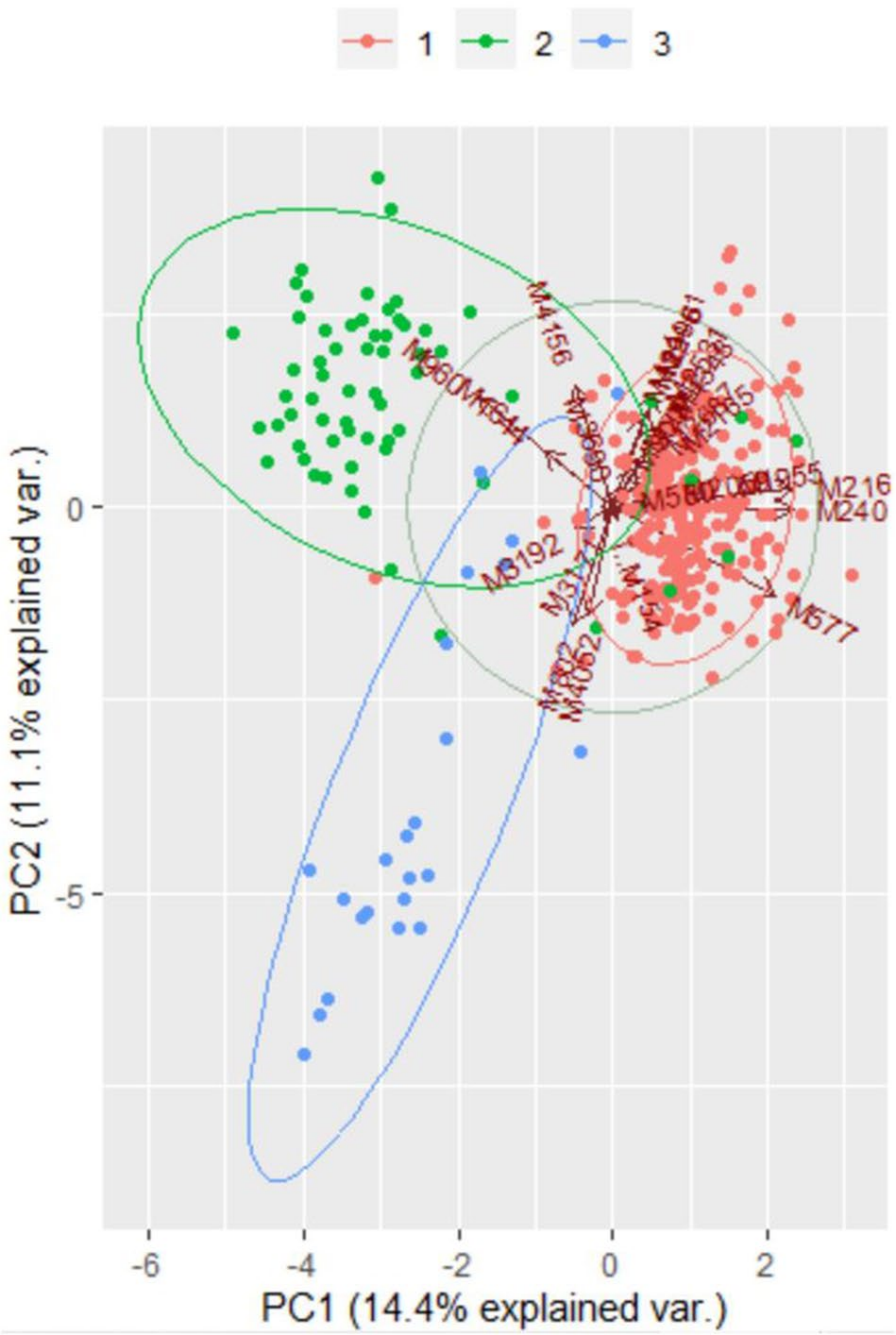
A Two-dimensional scatter plot of MTBC isolates from Sikkim, India based on principal component analysis. Based on eigenvalues, the first two components account for 14.4% and 11.1%, of the total variation of the entire dataset. The MTBC isolates were colour-coded depending upon their spoligotype lineage (Orange: Beijing; Green; CAS1_Delhi and Blue; T1). Software package R programme was used for analysis.

To visualize possible clustering of MTBC isolates according to spoligotypes, we reduced the multidimensional MIRU-VNTR data into a few principal components. The first two components were used to depict MTBC isolates in the biplot (Fig. 5). MTBC isolates belonging to Beijing, CAS1_Delhi, and T1 tend to form separate groups as can be seen in the bi plot.

### Random forests (RF), support vector machines (SVM) and artificial neural networks (ANN)

In this study, we aimed to predict three dominant spoligotype using RF, SVM and ANN. We used supervised learning i.e. the machine learning (ML) algorithm was first trained on a training data set (70% randomly selected data) to learn predictive patterns and subsequently applied to testing data set (30% data which was kept aside and not used for model training) for evaluation of classification accuracy, sensitivity and specificity. We used tenfold cross-validation for SVM and ANN models. The testing data sets are non-overlapping. Finally, k-ML models are generated. *K*-fold cross-validation helps in avoiding model overfitting and the metric calculations of model performance are calculated as mean over the k-folds. The model specifications of SVM analysis were: Number of independents is equal to 24 (i.e. 24-loci MIRU-VNTR), SVM type was C-classification, Kernel type was Radical Basis Function. Hyperparameter optimisation revealed that best parameters for Epsilon was = 0 and Cost = 4.The results of SVM analysis are given as a confusion matrix showing predicted and observed Spoligotypes of Beijing, CAS1-Delhi & T1 Spoligotypes for training data set and testing data set (Table 6). The sensitivity, specificity, and accuracy of SVM classification/prediction for the training data set and the Testing data set are given in Table 7. For testing dataset, the sensitivity of detecting Beijing Spoligotype of MTBC was 97.06%and the specificity was 100%. For CAS1-Delhi the sensitivity was 97.06%and specificity was 100%. For the detection of the T1 Spoligotype of MTBC, the sensitivity and specificity were 97.06%and 100%, respectively.

**Table 6:** Confusion matrix showing three major spoligotypes of MTBC from Sikkim based on actual spoligotyping conducted using reverse hybridization (row data). Predicted lineages (columns) are based on the support vector machine (SVM) and artificial neural network (ANN) analysis using 24-loci MIRU-VNTR profiles. Based on k-fold cross-validation.

Actual lineages			
	Beijing	CAS1-Delhi	T1
Predicted lineages			
Based on RF			
**Training data set**			
Beijing	181	0	0
CAS1-Delhi	0	44	0
T1	0	0	13
**Testing data set**			
Beijing	65	0	0
CAS1-Delhi	3	19	1
T1	0	0	9
Based on SVM			
**Training data set**			
Beijing	181	1	0
CAS1-Delhi	0	43	0
T1	0	0	13
**Testing data set**			
Beijing	66	0	0
CAS1-Delhi	2	19	2
T1	0	0	8
Based on ANN			
**Training data set**			
Beijing	180	4	0
CAS1-Delhi	0	38	0
T1	0	1	14
**Testing data set**			
Beijing	69	3	0
CAS1-Delhi	0	17	1
T1	0	0	8

**Table 7:** Showing performance measure, i.e. (accuracy, sensitivity and specificity) of random forest (RF)/support vector machine (SVM)/artificial neural network (ANN) analysis. Based on k-fold cross-validation. The training data set is based on 70% of MTBC isolates from Sikkim selected randomly and testing data set is remaining 30% of MTBC isolates from Sikkim which were not used for model training.

Model	Type of data set	Performance measure	Beijing*	CAS1-Delhi*	T1*
RF	Training	Sensitivity	100 (97.98–100)	100 (91.96–100)	100 (75.29–100)
		Specificity	100 (93.73–100)	100 (98.12–100)	100 (98.37–100)
		Accuracy	100 (98.46–100)	100 (98.46–100)	100 (98.46–100)
	Testing	Sensitivity	95.59 (87.64–99.08)	100 (82.35–100)	90 (55–99.75)
		Specificity	100 (88.06–100)	94.87 (87.39–98.59)	100 (95.85–100)
		Accuracy	96.91 (91.23–99.5)	95.88 (89.78–98.87)	98.97 (94.39–99.97)
SVM	Training	Sensitivity	100 (97.98–100)	97.73 (87.98–99.99)	100 (75.29–100)
		Specificity	98.25 (90.61–99.96)	100 (98.12–100)	100 (98.37–100)
		Accuracy	99.58 (97.68–99.99)	99.58 (97.68–99.99)	100 (98.46–100)
	Testing	Sensitivity	97.06 (89.78–99)	97.06 (89.78–99.64)	97.06 (89.78–99.64)
		Specificity	100 (88.06–100)	100 (80.06–100)	100 (88.06–100)
		Accuracy	97.94 (92.75–99.75)	97.94 (92.75–99.75)	97.94 (92.75–99.75)
ANN	Training	Sensitivity	99.43 (96.84–99.99)	92.5 (79.61–98.43)	100 (78.2–100)
		Specificity	98.18 (90.28–99.95)	99.47 (97.09–99.99)	99.07 (96.66–99.89)
		Accuracy	99.13 (96.88–99.89)	98 (95.59–99.52)	99.13 (96.88–99.89)
	Testing	Sensitivity	100 (78.2–100)	87.5 (67.64–97.34)	100 (54.07–100)
		Specificity	99.07 (96.66–99.89)	100 (95.55–100)	97.98 (92.89–99.75)
		Accuracy	99.13 (96.88–99.89)	97.14 (91.88–99.4)	98 (93.29–99.77)

*The values are in % and 95% confidence intervals are given in parenthesis for SVM/ANN.

A multilayer perceptron network was used for ANN. K-fold cross-validation was also performed for ANN analysis. The input layer consisted of 24 factors (24-MIRU-VNTR types). The number of units includes 24 (excluding the bias unit). The number of hidden layers was 1 and the number of units in the hidden layer were7 (excluding the bias unit). The activation function used was Hyperbolic Tangent. The output layers included one dependent variable for Spoligotypes (Beijing or CASI Delhi or TI). The activation function used was SoftMax and the error function was cross-entropy.

**Figure 6:**
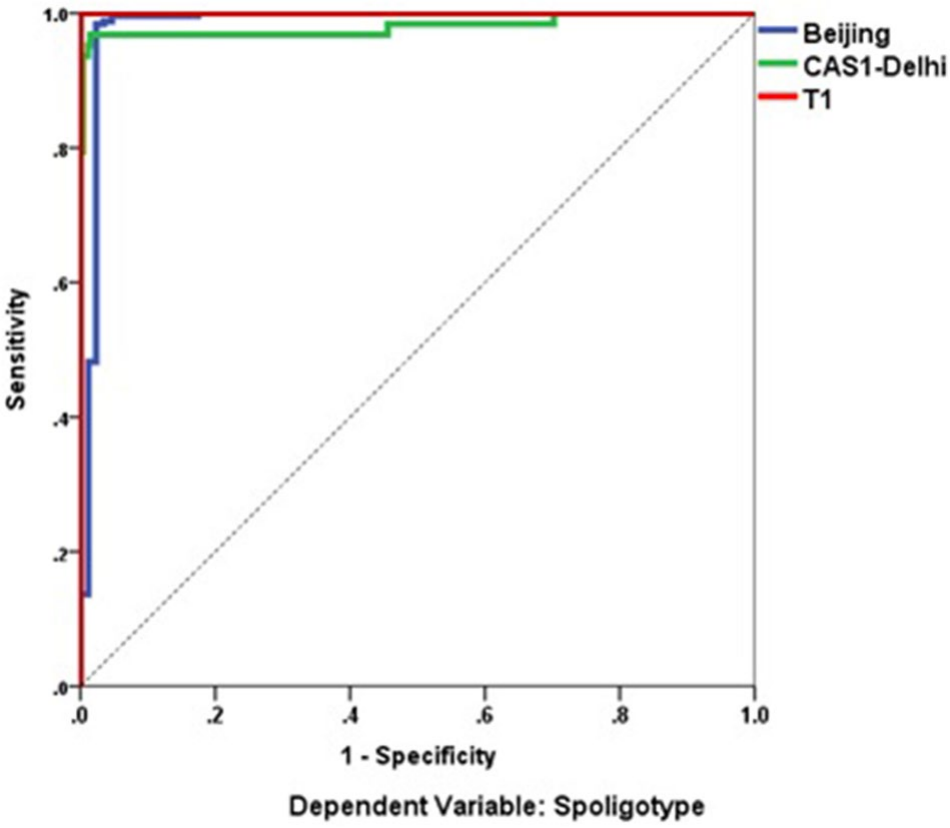
A Receiver operating characteristic (ROC) Curve and measured area under curve (AUC) showing, the classification performance of artificial neural network (ANN) at different levels of cut-offs (threshold levels). This sensitivity versus specificity plot shows the high performance of ANN in predicting spoligotype lineages. Software package R progamme was used for analysis.

**Figure 7:**
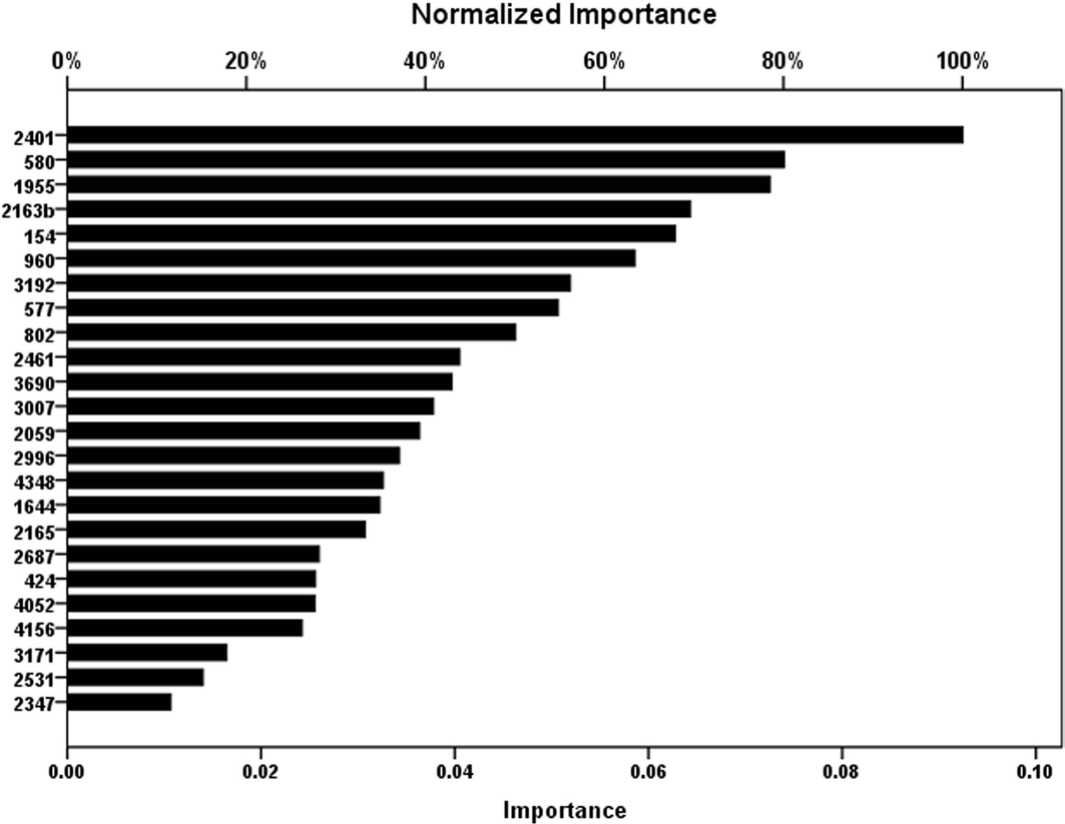
Importance value of independent variables (24 MIRU-VNTR loci) useful for predicting three dominant spoligotypes (Beijing orCAS1_Delhi, orT1) based on artificial neural network (ANN) analysis.

The result of tenfold cross-validation for predicting Beijing or CAS1-Delhi or T1 Spoligotype using Artificial Neural Network analysis for testing, training datasets are given in Table 6. The accuracy for the prediction of three dominant Spoligotypes (Beijing, CAS1-Delhi, and T1) in the testing data set was 97–99% (Table 7). The ROC analysis for ANN model for predicting Beijing, CAS1-Delhi and T1 MTBC Spoligotypes based on testing data set from Sikkim which were not used for model training (Fig. 6) shows sensitivity versus specificity graph i.e. classification performance for all possible cut-offs. The curves of Beijing, CAS1-Delhi, and T1 are quiet away from the 45° baseline indicating a more accurate and robust classification achieved by ANN. This interpretation is also supported by significantly high Area Under Curve (AUC) result. The 24-MIRU_VNTR independent variables ranked on the basis of their importance for prediction of MTBC spoligotype is given in importance chart (Fig. 7). The importance values of each independent (predictor) variable is computed based on training and testing samples as implemented in SPSS v.26 (https://www.ibm.com/in-en/analytics/spss-statistics-software). The normalized importance values are computed by dividing importance values by the largest importance value and expressed as percentage.

### Random forest analysis.

The optimal number of decision trees was found to be 3000 and optimal number of variables used at each split was found to be four. The out of bag (OOB) estimate of error of final tuned model based on training data set was 3.78%. The confusion matrix for training and testing data set is given in tables 7and 8, respectively.

### External validation and performance evaluation of SVM and ANN

We used MTBC dataset from different region (Assam) for validating of ML models. All ML models used in the present study viz., RF, SVM and ANN models were trained to predict Beijing or CAS1-Delhi or T1 Spoligotypes using data from MTBC isolates obtained from Sikkim. However, to validate the performance of RF, SVM & ANN models new data set used was based on MTBC data generated from Assam. The accuracy, sensitivity and specificity of RF, SVM and ANN models against external new data set are given in Table 8. The results show that RF is better classifier to predict Beijing or CAS1_Delhi or T1 strains of MTBC using MIRU-VNTR data.

**Table 8:** Showing performance measure, i.e. (accuracy, sensitivity and specificity) of random forest (RF)/support vector machine (SVM)/artificial neural network (ANN) analysis based on external database validation. The training data set is based on MTBC isolates from Sikkim and testing data set is based on MTBC isolates from a different geographical area (the state of Assam), which was not used for model training.

Model	Type of data set	Performance measure	Beijing*	CAS1-Delhi*	T1*
RF	Training	Sensitivity	95.89 (range 88.46–99.14)	95.35 (range 84.19–99.43)	93.75 (range 69.77–99.84)
		Specificity	94.92 (range 85.85–98.94)	100 (range 98.12–100)	100 (range 96.87–100)
		Accuracy	95 (range 90.37–98.31)	100 (range 98.46–100)	99 (range 95.86–99.98)
	Testing	Sensitivity	100 (97.98–100)	100 (91.96–100)	100 (75.29–100)
		Specificity	100 (93.73–100)	100 (98.12–100)	100 (98.37–100)
		Accuracy	100 (98.46–100)	100 (98.46–100)	100 (98.46–100)
SVM	Training	Sensitivity	95.89 (range 88.46–99.14)	93.02 (range 80.94–98.54)	75 (range 47–92.73)
		Specificity	91.53 (range 81.32–97.19)	94.38 (range 87.37–98.15)	100 (range 96.87–100)
		Accuracy	94.74 (range 88.41–97.35)	93.94 (range 88.41–97.35)	96.97 (range 92.42–99.17)
	Testing	Sensitivity	100 (97.98–100)	97.73 (87.98–99.94)	100 (75.29–100)
		Specificity	98.25 (90.61–99.96)	100 (98.12–100)	100 (98.37–100)
		Accuracy	99.58 (97.68–99.99)	99.58 (97.68–100)	100 (98.56–100)
ANN	Training	Sensitivity	98.63 (range 92.6–99.97)	83.72 (range 69.3–93.19)	87.5(range 61.65–98.45)
		Specificity	88.14 (range 77.07–95.09)	98.88 (range 93.9–99.97)	98.28 (range 93.91–99.79)
		Accuracy	93.94 (range 88.41–97.35)	93.94 (range 88.41–97.35)	96.97(range 92.42–99.17)
	Testing	Sensitivity	99.43 (96.86–99.99)	92.5 (76.61–98.43)	100 (78.2–100)
		Specificity	98.18 (90.28–99.95)	99.47 (97.09–99.99)	99.07 (96.66–99.89)
		Accuracy	99.13 (96.88–99.89)	98.25 (95.59–99.52)	99.13 (96.88–99.89)

*The values are in % and 95% confidence intervals are given in parenthesis for RF/SVM/ANN.

## Discussion

India has still the highest burden of TB despite intense national efforts to control and eliminate it (RNTCP, 2014). TB is difficult to control and eliminate in India probably due to its vast geographical and socio-economic diversity. Recent global studies have shown that high genotypic diversity of MTBC strains is an important factor in the pathogenesis of TB by affecting virulence, transmissibility, host response and the emergence of drug resistance^53^.

Recent advances in MIRU-VNTR profiling and spoligotyping methods have provided powerful tools to determine various MTBC strains circulating in TB patients and to understand transmission dynamics of tuberculosis in a region^31^. Till date, only limited studies have been conducted on 24-loci MIRU-VNTR and spoligotyping based method to characterize MTBC strains in India^31^. Our study based on 24-loci MIRU-VNTR and spoligotyping of 399 MTBC isolates provides the first insight into the population structure of MTBC isolates from the hill state of Sikkim. According to this study the *Beijing* spoligotype was found to be the most dominant Spoligotype responsible for tuberculosis transmission in Sikkim, followed by CAS1_Delhi. The Delhi/CAS Spoligotype is effectively confined to India, Western Asia and Eastern Africa^14^. The *Beijing* strains, first described by Van Soolingen et al., in the *Beijing* area in 1995^54^. The Beijing Spoligotype of MTBC is dominant in countries from Eastern Asia, Central Asia, Northern Asia and South-Eastern Asia although Beijing strains have also been reported from Austral Africa, Austral Asia, Southern Asia, Western Asia, North America, Central America, Northern Europe and Southern Europe. In India except NE region *Beijing*/*Beijing*-like strains of MTBC are less prevalent and their frequency ranges from 3 to 7%^35^. The Beijing strain of MTBC is more dominant in Sikkim, about 62.41% (present study) and 35.45% in Assam^19^. The dominance of *Beijing* genotype in Sikkim is a matter of great concern as this genotype has been associated with the high frequency of the drug resistance^4,55^; and treatment failure^56,57^. Moreover, the *Beijing* genotype is known to cause epidemic outbreaks in several countries because of their high adaptability and also this strain is considered to be less sensitive to BCG vaccination^58,59^. Our present study has revealed that Multiple Drug Resistant tuberculosis (MDR-TB) is more prevalent in Beijing strains 29.7% (n = 249), whereas in Non-*Beijing* strains of MTBC prevalence of MDR was 4.7% (n = 150) only. Multiple Drug Resistance thus appears to be associated with the *Beijing* strains in North Eastern region of India as it has been previously observed in other Southeast Asian countries like Vietnam, Thailand and also in South Africa^2,56,60–64^.

The predominance of *Beijing* isolates in Sikkim indicates that more attention is needed to be given to the TB control program in this region to prevent the spreading of this dominating genotype in the community. Recent studies have shown that the modern *Beijing* strains of MTBC are spreading throughout the world because of their high degree of transmission potential^65^ and BCG vaccination has been found to favour the positive selection of *Beijing* strains^66^.

In addition to *Beijing* family strains, we also identified strains belonging to other families such as CAS1_Delhi (15.79%), T1 (5.76%), Orphan (4.26%), CAS (1.75%), T4 (1.50%), CAS2, H3, LAM6 (1.00%), H1, MANU2, UNKNOWN (0.75%), EAI7-BGD2, EAI5 T3, T5 (0.50%), The Less frequent strains belonged to EAI3-IND, LAM9, URAL-2, X1 and X2 (0.25%). MIRU-VNTR profiling (24-loci) was more discriminatory (HGDI = 0.9999) of genotyping method as compared to spoligotyping method (HGDI = 0.59).

In this study we tried to predict the main spoligotypes of MTBC in Sikkim, India using 24-loci MIRU-VNTR profiles. Two-dimensional scatterplot of MTBC isolates indicates that 24-loci MIRU-VNTR data can group MTBC isolates according to their spoligotype (Fig. 4). These preliminary results encouraged us to explore the effectiveness of RF, SVM & ANN to predict dominant spoligotype of MTBC using 24-loci MIRU-VNTR profile. The results of testing data (unseen sample) clearly indicate that classification; accuracy rate for ANN was significantly high, followed by RF and SVM models. However, RF model turned out to be better predictor of MTBC spoligotype when new external data was used for testing. The major limitation of this study is small sample size for some Spoligotypes. Further studies are needed using more diverse samples from different geographical areas to validate these finding at global level. Nevertheless, this study has clearly shown the possible use of Artificial Intelligence in predicting Spoligotypes from 24-loci MIRU-VNTR profiles. The high-resolution molecular characterization of MTBC done in the present study gives us the first insight into the genotypic diversity of MTBC isolates from Sikkim, where MDR TB is emerging as an important public health concern. The results of the present study are interesting due to the high predominance of *Beijing* genotype. However, more elaborate longitudinal studies are needed to be undertaken in this region to understand the transmission dynamics of MTBC, and also to get an insight into the efficiency of the TB control program in Sikkim.

## Methods

### Bacterial culture, identification and DNA extraction

A total of 399 AFB positive sputum samples were collected from 2016 to 2018 from Sikkim and brought to the ICMR-Regional Medical Research Centre, North-East Region laboratory Dibrugarh, for culture, Drug Sensitivity Testing (DST) and molecular characterization of MTBC isolates. Biosafety level 3 was used for culture and DST, and BSL level 2 facility was used for molecular experiments. Modified Petroff's method was used to decontaminate sputum samples, and all the samples were subjected to culture on solid LJ media at 37 °C for 6–8 weeks. The *Mycobacterium* species identification was performed according to traditional microbiological and biochemical methods^43,67^and subsequently compared with their respective spoligotyping and MIRU-VNTR patterns.

### Drug sensitivity testing (DST)

DST was done using the proportion method^67^for all first-line anti-TB drugs like rifampicin (RIF), isoniazid (INH), streptomycin (STR), ethambutol (EMB) and pyrazinamide (PZA).

### DNA isolation

DNA was extracted from fresh cultures by the cetyl-trimethyl ammonium bromide (CTAB) method^68^.

### Spoligotyping

For the detection of presence or absence of 43 spacers was done on all isolates as described by Kamerbeek, et al^69^. using a commercially available kit (ISOGEN BIOSCIENCES, BV, Maarsen the Netherlands now Ocimum Biosolutions). Briefly, the direct repeat (DR) region was amplified with primer pair Dra, 5′-GGTTTTGGGTCTGACGAC-3′ (biotinylated 5′ end) and DRb, 5′-CCGAGAGGGGACGGAAAC-3′. The DNA amplification was carried out in GENEAMPPCR system 9700 of Applied Biosystems. The amplified PCR products were hybridized with nitrocellulose membrane having covalently linked 43 spacer oligonucleotides following the standard procedure^69^. The hybridized fragments were detected using an enhanced chemiluminescence system (GE Healthcare, UK Ltd., Buckinghamshire, UK) and subsequent exposure in X-ray film in darkroom^70^. The spoligotypes were initially reported as 43 digits binary representation of 43 spacers; one was scored for positive hybridization and zero for no hybridization.

### MIRU-VNTR typing

MIRU-VNTR typing was performed by amplifying 24 hypervariable MIRU loci of all 399 isolates of MTBC from Sikkim. These 24 MIRU loci used for typing in this study are MIRU02, Mtub04, ETRC, MIRU04, MIRU40, MIRU10, MIRU16, Mtub21, MIRU20, QUB11b, ETRA, Mtub29, Mtub30, ETRB, MIRU23, MIRU24, MIRU26, MIRU27, MTUB34, MIRU31, Mtub39, QUb26, QUB4156 and MIRU39. The details of primer pairs and PCR reaction conditions are given by Supply et al., 2006^46^*.* The PCR products' fragment sizes were determined in the LAB CHIP (Caliper life sciences Inc., USA) or agarose gel electrophoresis. The copy number of the tandem repeats was calculated as a function size of the PCR product and interpretation based on the reference table^46^. In doubtful cases, the experiment was repeated for confirmation. For quality control, *Mycobacterium tuberculosis* H37Rv and one *Beijing* strain were used in every batch of the experiment.

### Genotype analysis and comparison with databases

Web tools MIRU-VNTR*plus *(https://www.miru-vntrplus.org/)* and SITVIT2 *(http://www.pasteur-guadeloupe.fr:8081/SITVIT2/) were used for assignment of MTBC species, Spoligotypes, and genotypes by comparing with international reference database strains^45,70,71^. Spoligotypes were identified by a similarity search in MIRU-VNTR*plus* and SITVIT2. As on 3^rd^ April 2020, the SITVIT2 database contains 1,11,635 entries from 177 countries. In this database, the spoligotypes are designated as Spoligotype International Type (SIT) if isolates share them from two or more patients, and if a spoligotype is from a single patient, it is designated as orphan^70^.

Phylogenetic genetic analysis of 399 isolates and the international reference strains was done using Neighbour joining (NJ) tree method based on combined analysis of spoligotypes & 24-MIRU-VNTRs implemented by MIRU-VNTRplus web tool. A Minimum Spanning Tree (MST) using 24-loci MIRU-VNTR dataset was also constructed for *Beijing* strains (n = 249) and Non-Beijing strains (n = 150) to determine their Clonal Complexes (CC). We allowed single-locus variants (SLVs) to be included in clonal complexes and identical patterns of MIRU-VNTR. Clonal Complexes identified genetically closely similar strains sharing common transmission link^41,72^*.* Unknown spoligotypes/orphan strains were subjected to phylogenetic tree analysis using the Neighbour Joining (NJ) tree and categorical coefficient to predict these isolates' Spoligotype. The discriminatory power of spoligotyping and MIRU-VNTR typing system was calculated using the Hunter Gaston Discriminatory Index (HGDI)^73^.

Where *N* = the total number of strains in the sample population, *S* is the total number of types described, and the *NJ* tree is the number of strains belonging to the j^th^ type.

RStudio version 1.3.1093 and R version 4.0.3 was used for Principal components analysis (PCA). Built in R function ‘prcom’ and libraries ‘devtools’, ‘ggplot2′,‘plyr’, ‘scales’ and ‘grid’ were also used. First two PCs were used for plotting MTBC isolates (n = 335) in 2-D scatter-plot to get an idea if these MTBC strains tended to group according to their spoligotype (Beijing, CAS1-Delhi or T1). The MTBC isolates were colour coded by Spoligotype type to visualize possible clustering in three groups.

### Dataset preparation

Target input variable was categorical representing three classes of MTBC Spoligotypes viz., Beijing, CAS1_Delhi and T1 and was encoded as 1, 2 or 3 representing three classes. Independent input variables were 24 in number, and all were numeric. Numeric variables were normalized to have values ranging between 0 and 1. No data was missing.

### Random forests, support vector machines and artificial neural networks

In the present study, artificial intelligence-based machine learning methods such as random forests, support vector machines and artificial neural networks^74–79^ were used to predict dominant spoligotypes of MTBC using MIRU-VNTR data. Random forest is a robust supervised classification/regression machine learning technique. It is an ensemble classifier based on constructing 100 s of decision trees at training time. The bootstrap samples are used to grow numerous decision trees, and a random sample of independent predictors are used at each node. We used two parameters in RF for tuning viz., ‘ntree’ number of trees to grow and ‘mtry” number of variables to select at a node split. RF model is robust and does not overfit the training data. RF analysis was conducted using R package (V 4.0.3). Libraries ‘caret’, ‘lattice’ & ‘ggplot2′ were used for RF analysis.

Support Vector Machines (SVM) are advanced nonparametric machine learning data mining techniques based on supervised and kernel-based methods. SVM is used for classification, prediction and regression problems. Learning in the SVMs is achieved by finding an optimal linear hyperplane using appropriate kernel functions, maximizing the margin between the classes. The classification can be binary or multiclass. Prediction of spoligotype using 24-loci MIRU-VNTR profile of MTBC is an example of the multiclass classification task. In the present study, the data set of 335 MTBC isolates from Sikkim having information on three dominant spoligotypes and 24-loci MIRU-VNTR profiles. We used *k-*fold cross-validation (*10-*fold for Beijing or CAS1-Delhi and T1 MTBC Spoligotype) to test the performance of the model. The single dependent variable was categorical (‘1’ for Beijing ‘2’ for CAS1/Delhi and ‘3’ for T1) and 24 independent variables (24-loci MIRU-VNTR profile) were numeric. We used ‘caret’ and ‘e1071’ libraries in R for SVM analysis. We tested ‘radial’, ‘linear’, polynomial’ and ‘sigmoid’ kernel functions to determine best function suitable for classifying three dominant MTBC Spoligotypes. The optimal value for ‘Epsilon’ and ‘Cost’ were determined using ‘tune’ library. Artificial Neural Networks (ANN) are currently popular and powerful machine learning tools that are biologically inspired computational models that imitate brain neurons and solve complex problems. The ANN typically consists of the three-layered network (the input layer, the hidden layer and the output layer) consisting of artificial neurons or nodes and interconnected by connections (synaptic weights). ANN require training data (supervised learning algorithm) for model building. The dependant and independent variables are given as input (training phase) that information will be used for the system to learn using the back-propagation learning algorithm to predict outputs. The Multilayer Perceptron (MLP) was used to build ANN. K-fold cross-validation was also used for the evaluation of ANN. We used SPSS v26 for ANN analysis. SPSS software has the provision to manually choose parameters such as number of hidden layers, number of units in hidden layers, activation functions (hyperbolic tangent or sigmoid), and output layer activation functions like identity, SoftMax, hyperbolic tangent & sigmoid. Instead, we opted for automatic architecture selection to select optimal parameters with a number of hidden layers one to fifty. The accuracy of the ANN model was best as revealed by ROC analysis.

Sensitivity, specificity, and accuracy were calculated to measure the performance of SVM and ANN predictions. Sensitivity was measured by the formula TP/(TP + FN), specificity was measured as TN/(FP + TN), and accuracy by (TP + TN)/(TP+TN+FP+FN) where TP, TN, FN and FP represent true positive, true negative, false negative and false positive, respectively. The Receiver Operating Characteristic Curve (ROC) analysis was also used to determine ANN classifiers' performance, where x-axis represents 1-specificity and the y-axis represents sensitivity and the value ranges between 0.0 and 1.0.

For external validation, 132 isolates of MTBC collected from different geographical areas (the state of Assam) were also processed for spoligotyping and 24-loci MIRU-VNTR typing. To check for overfitting, we used blind external data (obtained from MTBC isolates from Assam) to evaluate model performance of all ML methods i.e. RF, SVM and ANN.

Excel 2016 of Microsoft office was used for calculations related to performance measure of RF, SVM and ANN classifiers. This was done by generating confusion matrices generated for training, testing and external new data sets.

### Logistic regression analysis

Binary logistic regression analysis was used to find the association between multiple drug resistance (MDR) status and MTBC Spoligotype. The dependant variable used was MDR status of MTBC isolate and the independent variable used was whether the MTBC isolate belonged to Beijing or non-Beijing Spoligotype. The Wald test was used to find statistical significance of Independent variable. The strength of the Association between MDR and Spoligotype was determined using the odds ratio and 95% confidence interval of the odds ratio.

### Ethics approval and consent to participate

This study was approved by the Ethical Committee of ICMR-Regional Medical Research Centre, North-East Region, Dibrugarh. All processes were performed in accordance with the related regulations and guidelines. Written informed consent was obtained from all the participants or their guardians in the case of minors who provided sputum samples. Patients found positive for AFB were referred to the nearest DOTS centre for treatment.

## Supplementary Information

Supplementary Legends.

Supplementary Figure 1.

Supplementary Figure 2.

Supplementary Figure 3.
