# Supplementary material for: Molecular diversity of Mycobacterium tuberculosis complex in Sikkim, India and prediction of dominant spoligotypes using artificial intelligence
Source: Sci Rep. 2021 Apr 1;11:7365. doi: 10.1038/s41598-021-86626-z (PMC8016865; doi:10.1038/s41598-021-86626-z)
Supplement: Supplementary file 1 — Supplementary Legends. [file 41598_2021_86626_MOESM1_ESM.docx]

**Title Page**

**Molecular diversity of *Mycobacterium tuberculosis* complex in Sikkim, India and prediction of dominant spoligotypes using artificial intelligence**

Kangjam Rekha Devi^1^, Jagat Pradhan^2^,Rinchenla Bhutia^2^, Peggy Dadul^3^, Atanu Sarkar^1^, Nitumoni Gohain^1^, Kanwar Narain^1^

**Authors affiliations**

^1^Indian Council of Medical Research(ICMR)-Regional Medical Research Centre, N.E. Region, Post Box #105, Dibrugarh 786 001, Assam, India;

^2^Revised National Tuberculosis Control Program(RNTCP), Sikkim;

^3^State Tuberculosis Control Society, Department of Health Care, Human Services & Family Welfare, Gangtok Sikkim;

**Corresponding Author.** Dr. K. Narain, Director, ICMR-Regional Medical Research Centre, N.E. Region, Post Box #105, Dibrugarh 786 001, Assam, India;

**E-mail address.** kanwar_narain@hotmail.com

**Supplementary Fig. 1**

Neighbour-Joining (NJ) tree showing the phylogenetic relationship of orphan strains of MTBC from Sikkim with reference MTBC isolates available at the MIRU-VNTR*plus* database (<https://miru-vntrplus.org/MIRU/index.faces>). The NJ tree was constructed using spoligotyping and 24-loci MIRU-VNTR data. MIRU-VNTR alleles and spoligo-patterns for all MTBC isolates are given along with the NJ tree.

**Supplementary Fig. 2**

Neighbour-Joining (NJ) tree showing the phylogenetic relationship of 249 Beijingisolates of MTBC from Sikkim with reference MTBC isolates available at the MIRU-VNTR*plus* database (<https://miru-vntrplus.org/MIRU/index.faces>). The NJ tree was constructed using spoligotyping and 24-loci MIRU-VNTR data. MIRU-VNTR alleles and spoligo-patterns for all 249 Beijing MTBC isolates from Sikkim are given along with the NJ tree.

**Supplementary Fig. 3**

Neighbour-Joining (NJ) tree showing the phylogenetic relationship of 150 non-Beijingisolates of MTBC from Sikkim with reference MTBC isolates available at the MIRU-VNTR*plus* database (<https://miru-vntrplus.org/MIRU/index.faces>). The NJ tree was constructed using spoligotyping and 24-loci MIRU-VNTR data. MIRU-VNTR alleles and spoligo-patterns for all 150 non-Beijing MTBC isolates from Sikkimare given along with the NJ tree.
