# Supplementary figures and images for: Molecular diversity of Mycobacterium tuberculosis complex in Sikkim, India and prediction of dominant spoligotypes using artificial intelligence
Source: Sci Rep. 2021 Apr 1;11:7365. doi: 10.1038/s41598-021-86626-z (PMC8016865; doi:10.1038/s41598-021-86626-z)

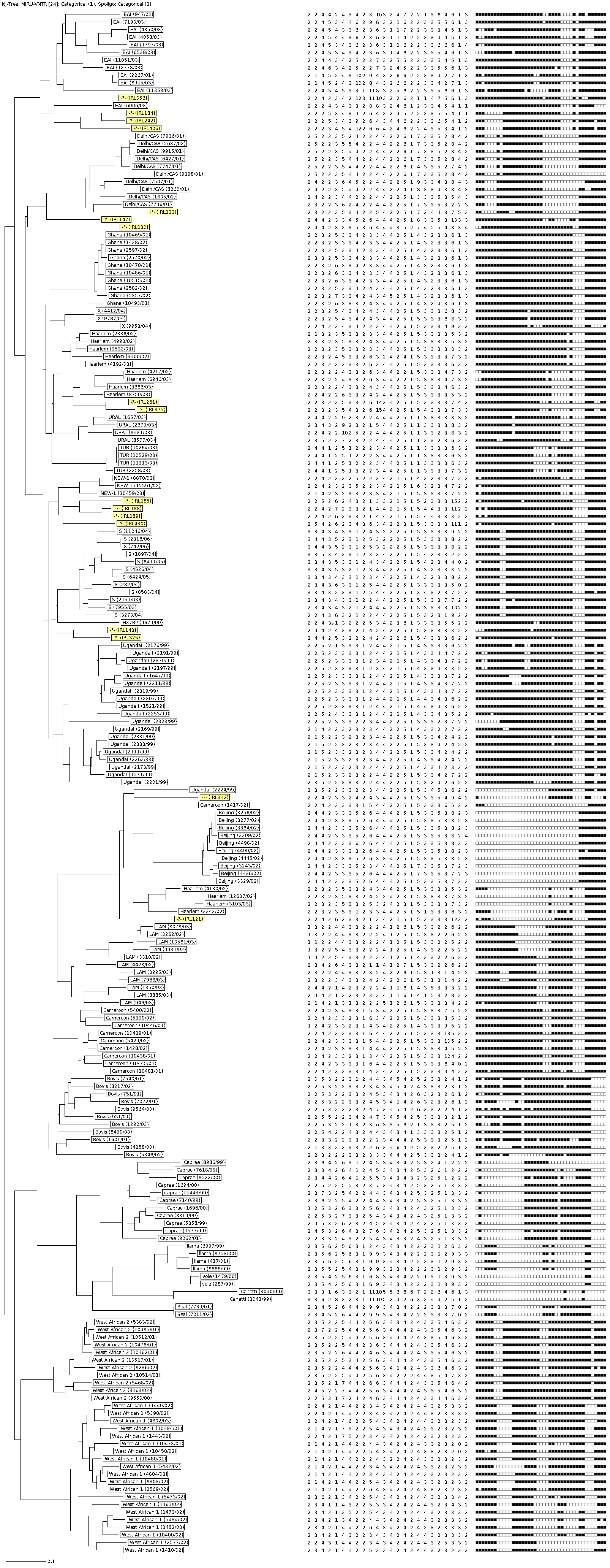

Supplement: Supplementary file 2 — Supplementary Figure 1. [file 41598_2021_86626_MOESM2_ESM.png]

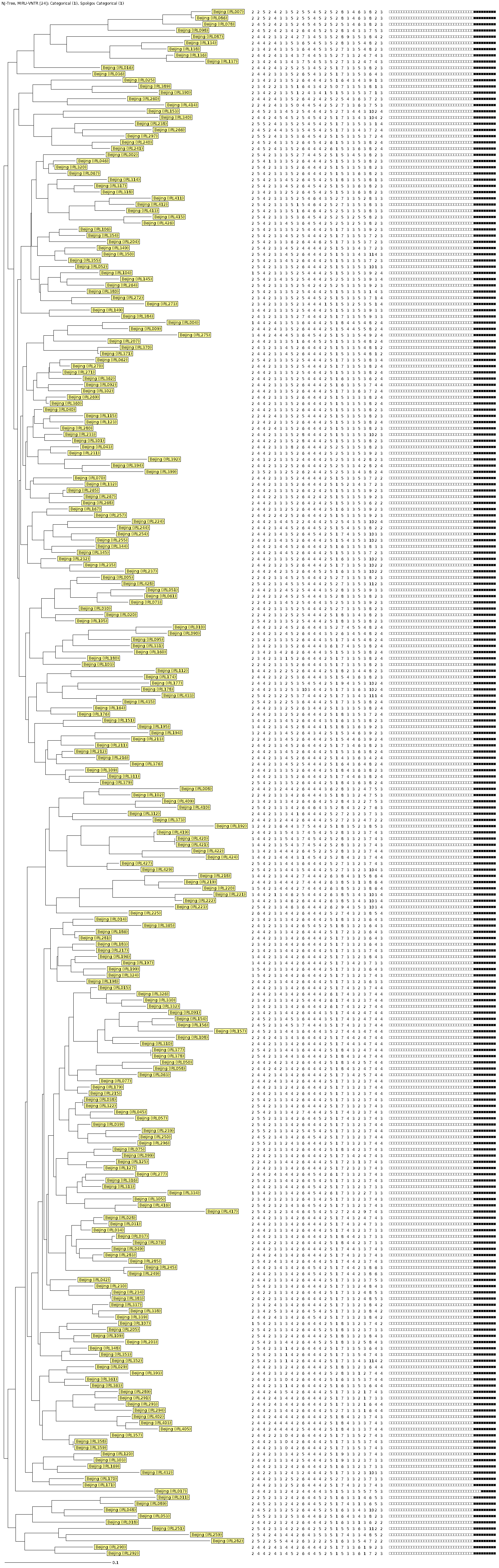

Supplement: Supplementary file 3 — Supplementary Figure 2. [file 41598_2021_86626_MOESM3_ESM.png]

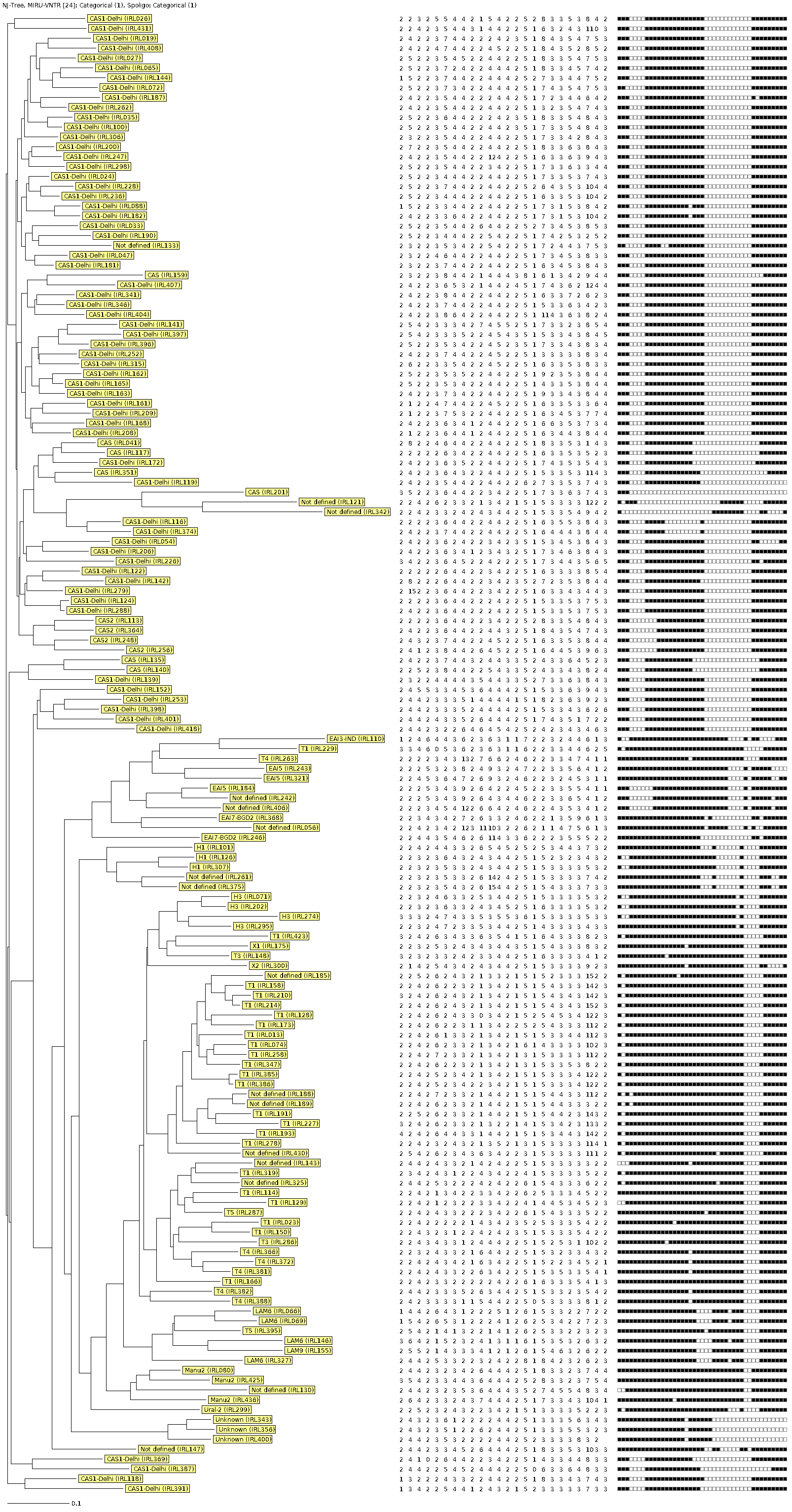

Supplement: Supplementary file 4 — Supplementary Figure 3. [file 41598_2021_86626_MOESM4_ESM.png]
